# Identification of functional pathways for regenerative bioactivity of selected renal cells

**DOI:** 10.1186/s13287-022-02713-6

**Published:** 2022-02-17

**Authors:** Wei Sha, Timothy Bertram, Deepak Jain, Cory Brouwer, Joydeep Basu

**Affiliations:** 1 Bioinformatics Services Division, Department of Bioinformatics and Genomics, University of North Carolina at Charlotte, 150 Research Campus Drive, Ste. 3333, Kannapolis, NC 28081 USA; 2 Prokidney, LLC, Winston-Salem, NC 27103 USA

**Keywords:** Cell therapy, Tissue engineering, Chronic kidney disease, Preclinical animal model, Stem cell, Transcriptomics, Mechanism of action, Selected renal cells, Potency assay

## Abstract

**Background:**

Selected renal cells (SRC) are in Phase II clinical trials as a kidney-sourced, autologous, tubular epithelial cell-enriched cell-based therapy for chronic kidney disease (CKD). In preclinical studies with rodent models of CKD, SRC have been shown to positively modulate key renal biomarkers associated with development of the chronic disease condition.

**Methods:**

A comparative bioinformatic analysis of transcripts specifically enriched or depleted in SRC component sub-populations relative to the initial, biopsy-derived cell source was conducted.

**Results:**

Outcomes associated with therapeutically relevant bioactivity from a systematic, genome-wide transcriptomic profiling of rodent SRC are reported. Key transcriptomic networks and concomitant signaling pathways that may underlie SRC mechanism of action as manifested by reparative, restorative, and regenerative bioactivity in rodent models of chronic kidney disease are identified. These include genes and gene networks associated with cell cycle control, transcriptional control, inflammation, ECM–receptor interaction, immune response, actin polymerization, regeneration, cell adhesion, and morphogenesis.

**Conclusions:**

These data indicate that gene networks associated with development of the kidney are also leveraged for SRC regenerative bioactivity, providing evidence of potential mechanisms of action.

**Supplementary Information:**

The online version contains supplementary material available at 10.1186/s13287-022-02713-6.

## Background

Chronic kidney disease (CKD) represents a significant and increasing healthcare issue throughout the world. Although whole kidney transplantation remains the standard of care, the CKD patient population advancing to end-stage renal disease (ESRD) requiring dialysis or transplant is much greater than the number of acceptable donor kidneys [1]. Regenerative medicine and tissue engineering methodologies may provide additional therapeutic possibilities for CKD patients. From a manufacturing and product development perspective, primary cell types are preferable when compared to stem and progenitor cell populations, owing to considerable streamlining of isolation, expansion, maintenance, and characterization conditions (reviewed by [2, 3]).

Several studies on regeneration of renal architecture and function following acute kidney injury point to tubular epithelial cells as central in restoration of renal functionality [4, 5]. Starting with an autologously sourced renal tissue biopsy, tubular cell-enriched populations can be separated from other kidney cell types on the basis of differential buoyant density using density gradient centrifugation [6]. Direct injection of these bioactive renal cell sub-populations in the rat 5/6N_*x*_ model of CKD resulted in extended survival and enhancement of renal function for six months post-treatment [7]. The mechanism(s) of action by which this observed regenerative bioactivity is mediated may include secreted protein factors and miRNAs delivered via exosomes [8]. Other documented mechanisms include neo-nephrogenesis at the site of SRC delivery [9]. However, elucidation of the mechanisms of action of this and other cell-based therapeutics is not a straightforward problem and is best approached with multiple methodologies.

In the current manuscript, the specific aim is to apply bioinformatics methodologies to compare SRC component cellular sub-fractions against each other and against pre-gradient, unfractionated renal cells. Transcriptional networks and signaling pathways that are functionally relevant to the observed regenerative bioactivity associated with each of the post-gradient subpopulations are identified. These sub-populations have been nominally designated as B1–B5 in preclinical functional evaluations of SRC therapeutic potential in rodent models of CKD. KEGG pathways and Gene Ontology (GO) categories that are significantly up- or down-regulated in each of sub-fractions B1–B5 in comparison with pre-gradient fractionation (PreG) are reported. The significance of these changes in gene expression in understanding the mechanism of action of the observed reparative, restorative, and regenerative bioactivity of SRC is discussed.

## Materials and methods

### Isolation and preparation of rodent renal cell populations for genomic analysis

The preparation of selected bioactive primary renal cells from whole rat kidney has been previously described in detail [6, 7, 10]. Briefly, whole kidneys were harvested from 5-week-old male Lewis rats (Hilltop Labs, Scottsdale, PA, USA) and kidney tissue was dissociated enzymatically in a buffer containing 4.0 units/mL dispase (Stem Cell Technologies, Inc., Vancouver, BC, Canada) and 300 units/mL collagenase IV (Worthington Biochemical, Lakewood, NJ, USA). Red blood cells and debris were removed by centrifugation through 15% iodixanol (OptiPrep®, Axis Shield, Norton, MA, USA). Primary renal cells were seeded onto tissue culture-treated polystyrene plates (NUNC, Rochester, NY, USA) and cultured in 50:50 media, a 1:1 mixture of high-glucose Dulbecco’s modified Eagle medium (DMEM):Keratinocyte Serum Free Medium (KSFM) containing 5% Fetal Bovine Serum (FBS (Hyclone, Logan, UT, USA), 2.5 µg EGF, 25 mg Bovine Pituitary Extract (BPE), 1X ITS (insulin/transferrin/sodium selenite medium supplement), and antibiotic/antimycotic (all from Invitrogen, Carlsbad, CA, USA). Prior to post-culture cell separation, primary renal cell cultures were transferred from atmospheric oxygen conditions (21%) to a more physiologically relevant low-oxygen (2%) environment for 24 h, to improve cell separation efficiency.

Separation of primary renal cell cultures, prepared as 75 × 10^6^ cells in 2 mL unsupplemented KSFM (uKSFM), was performed by centrifugation through a four-step iodixanol (OptiPrep; 60% w/v in uKSFM) density gradient layered specifically for rodent (16%, 13%, 11%, and 7%) in 15-mL conical polypropylene tubes and centrifuged at 800 × g for 20 min at room temperature (without brake). After centrifugation, cellular sub-fractions were extracted from the gradient via pipette and collected as 4 distinct bands (B1–B4) and a pellet (B5). All bands were washed 3 times with sterile phosphate-buffered saline (PBS) prior to use. A pre-gradient sample (“PreG”) and whole kidney tissue sample (“Macro”) were collected from each rodent for comparative purposes. Culture conditions used for each rodent cell population are summarized in Table 1. RNA for genome-wide transcript analysis was prepared using Qiagen’s RNA isolation kit (Germantown, MD, USA) according to the manufacturer’s instructions. Two micrograms of RNA from each sample normalized as per Table 2 was used for genome-wide transcript analysis on the Affymetrix GeneChip Rat Genome 230 2.0 Array (Wake Forest University Health Sciences Microarray Core Facility, Winston-Salem, NC, USA).

**Table 1:** Culture conditions and gradient load

Cell prep	Seeding density	Culture time	Final confluency (%)	Gradient load
RK086	17.5 e^6^/flask	3d 21% O_2_ 1d 2% O_2_	100	72.8 e^6^
RK087	15 e^6^/flask	2d 21% O_2_ 1d 2% O_2_	85	91 e^6^
RK097	19.3 e^6^/flask	2d 21% O_2_ 1d 2% O_2_	85	92.5 e^6^

*Gradient load* refers to the number of PreG cells loaded onto the iodixanol gradient for separation into SRC component sub-populations B1–B5

**Table 2:** RNA concentration and normalization

		RNA normalization				
		Fraction	Symbol	ng/ul	Vol, 2 µg	Norm 20 µl
1	RK086	3812	PreG	412.19	4.852	15.148
2		3813	B1	511.62	3.909	16.091
3		3814	B2	460.28	4.345	15.655
4		3815	B3	284.08	7.040	12.960
5		3816	B4	163.64	12.222	7.778
6		3817	Pellet	354.38	5.644	14.356
7	RK087	3821	Macro	213.05	9.387	10.613
8		3825	PreG	301.08	6.643	13.357
9		3826	B1	363.74	5.498	14.502
10		3827	B2	351.53	5.689	14.311
11		3828	B3	370.35	5.400	14.600
12		3829	B4	387.13	5.166	14.834
13		3830	Pellet	136.67	14.634	5.366
14	RK097	4692	Macro	125.76	15.903	4.097
15		4697	PreG	379.67	5.268	14.732
16		4698	B1	366.56	5.456	14.544
17		4699	B2	420.82	4.753	15.247
18		4700	B3	439.3	4.553	15.447
19		4701	B4	350.43	5.707	14.293
20		4702	Pellet	167.94	11.909	8.091

RNA has been normalized by resuspending a total of 2 µg of RNA in a total volume of 20 µl. In Table 2, the column “vol, 2 µg” lists the volume of the RNA preparation representing a total of 2 µg of RNA. The column “Norm 20 µl” lists the volume of additional buffer to be added to make up a final volume of 20 µl

### Data analysis

Affymetrix GeneChip data were normalized using RMA [11]. The gene expression profile from each fraction (B1, B2, B3, B4, B5) was compared to the gene expression profile in PreG by using paired t test. Differentially expressed genes were detected by paired t test at *P* < 0.05 and were then analyzed by DAVID (http://david.abcc.ncifcrf.gov) to detect Gene Ontology (GO) categories and KEGG Pathways that were significantly different between each fraction and PreG. For GO analysis, we used GO-BP-FAT, which is GO biological process categories provided by DAVID to minimize redundancy and increase specificity in GO terms. Bonferroni adjustment was applied to *P* values generated from GO and KEGG pathway analysis for multiple testing correction. KEGG pathway plots were generated using Pathview in Bioconductor [12]. Gene networks for GO categories were generated using QIAGEN’s Ingenuity Pathway Analysis (IPA®, QIAGEN Redwood City, USA, www.qiagen.com/ingenuity).

## Results

The manufacture of SRC is a linear process, with PreG cells isolated directly from source kidney tissue biopsies being used as starting material. Gradient sub-fractionation represents the processing stage that results in multiple sub-populations of cells (B1–B5). These sub-populations have clinically relevant functional properties in rodent models of chronic kidney disease that differ markedly from the initial PreG population [6, 7, 10]. This manuscript specifically aims to define how the final manufacturing sub-populations B1–B5 differ from the source material (PreG) by the use of genome-wide transcriptional profiling.

### Sub-fraction B1: GO/KEGG categories cell cycle, transcriptional control, and inflammation altered relative to PreG

GO analysis of the B1 sub-population relative to PreG shows that the GO categories associated with *regulation of the cell cycle* (*P* = 9.40E−04), *cell division* (*P* = 9.81E−05), and *cell cycle phase* (*P* = 0.016) were significantly down-regulated in B1 (Table 3). Similar outcomes were also generated by KEGG pathway analysis (Table 3; Additional file 1: Figure S1). As shown in the pathway map, the down-regulated genes are distributed in each phase of the cell cycle, including cell growth (G1, G2), DNA replication (S), and mitosis (M) (Additional file 1: Figure S1). Key cell cycle regulatory proteins subject to negative regulation in B1/PreG include CDK2, CDK7, CYCE, CDC45, ORC, CHK1, CHK2, MAD2, CDC20, and APC. Taken together, these proteins are instrumental in regulating each step of the mitotic sequence [13–15].

**Table 3:** GO categories and KEGG pathways that were significantly up- or down-regulated in B1 by comparing to PreG

	Category	Term	Raw_*P*	Bonferroni adjusted *P*
Up-regulated	GO_BP_FAT	GO-0019221: cytokine-mediated signaling pathway	7.12E−06	0.022732
		GO-0022610: biological adhesion	1.42E−05	0.044785
		GO-0007155: cell adhesion	1.42E−05	0.044785
	KEGG	rno04142:Lysosome	2.57E−06	4.44E−04
Down-regulated	GO_BP_FAT	GO-0051301: cell division	3.24E−08	9.81E−05
		GO-0007049: cell cycle	3.11E−07	9.40E−04
		GO-0045449: regulation of transcription	4.95E−07	0.001496
		GO-0022403: cell cycle phase	5.32E−06	0.015942
		GO-0051252: regulation of RNA metabolic process	1.51E−05	0.04449
	KEGG	rno03040:Spliceosome	1.35E−05	0.00234
		rno04110:Cell cycle	2.45E−04	0.041792

GO/KEGG categories significantly up-regulated in B1/PreG include *cytokine-mediated signaling pathway* (*P* = 0.023) (Additional file 1: Figure S2) which is involved in the immune response, and *lysosome* (*P* = 4.44E−04) which is involved in the digestion of foreign material and cell waste. *Cell adhesion* (*P* = 0.045) was also up-regulated (Table 3). Up-regulated pro-inflammatory cytokines identified in Additional file 1: Figure S2 include TNF, IL1A, CX3CL1, CCL2 [16]. Other pro-inflammatory or pro-adipogenic markers up-regulated in B1/PreG include LEPR and GAB1 [17].

### Sub-fraction B2: ECM–receptor interaction is sole GO/KEGG classifier altered relative to PreG

The transcriptomic profile of B2 was found to be similar to PreG at the pathway level. No GO/KEGG categories were identified as significantly up-regulated. Only one pathway, *ECM–receptor interaction* (rno04512), was found to be significantly down-regulated (*P* = 0.039). Numerous ECM proteins, including collagen, laminin, reelin, tenascin, vitronectin, and thrombospondin (THBS), were significantly down-regulated in B2 (Additional file 1: Figure S3) relative to PreG. In contrast to the negatively regulated transcriptomic profile observed with ECM-associated genes, RHAMM expression was specifically up-regulated in B2/PreG (fold change = 1.114, *P* = 0.030) (Additional file 1: Figure S3).

### Sub-fraction B3: GO/KEGG categories immune response and regulation of actin polymerization altered relative to PreG

KEGG pathway analysis found categories related to *complement and coagulation cascades* are significantly up-regulated in B3/PreG (*P* = 0.008) (Table 4; Additional file 1: Figure S4). Up-regulated genes include complement component genes, such as C2, C3, and C4, and coagulation-related genes, such as fibrinogen (FG) and coagulation factor XIII (F13) (Additional file 1: Figure S4). Consistent with KEGG analysis, GO analysis also found a significant increase in the *humoral immune response* (*P* = 0.025) (Table 4; Additional file 1: Figure S5). Several GO categories related to the *regulation of actin polymerization* and *actin filament length* were significantly down-regulated in B3 (Table 4; Additional file 1: Figures S6, S7).

**Table 4:** GO categories and KEGG pathways that were significantly up- or down-regulated in B3 by comparing to PreG

	Category	Term	Raw_*P*	Bonferroni adjusted *P*
Up-regulated	GO_BP_FAT	GO:0043112 ~ receptor metabolic process	2.14E−06	0.005782
		GO:0007242 ~ intracellular signaling cascade	3.59E−06	0.009661
		GO:0006959 ~ humoral immune response	9.21E−06	0.024598
		GO:0007167 ~ enzyme linked receptor protein signaling pathway	1.89E−05	0.049871
	KEGG	rno04610:Complement and coagulation cascades	6.60E−05	0.008351
Down-regulated	GO_BP_FAT	GO:0008064 ~ regulation of actin polymerization or depolymerization	2.42E−06	0.00522
		GO:0030832 ~ regulation of actin filament length	2.42E−06	0.00522
		GO:0030833 ~ regulation of actin filament polymerization	8.68E−06	0.018633

### Sub-fraction B4: GO/KEGG categories regeneration and cell adhesion altered relative to PreG

Consistent with established therapeutic bioactivity in vivo, multiple regeneration-related GO categories, including *vasculature development* (*P* = 7.76E−07), *blood vessel development* (*P* = 1.36E−06), *blood vessel morphogenesis* (*P* = 1.76E−06), *angiogenesis* (*P* = 1.82E−06) and *response to wounding* (*P* = 1.35E−05) were found to be significantly up-regulated in B4 over PreG (Table 5).

**Table 5:** GO categories that were significantly up-regulated in B4 by comparing to PreG

GO-BP-FAT term	Raw *P*	Bonferroni adjusted *P*
GO:0001944 ~ vasculature development	2.61E−10	7.76E−07
GO:0001568 ~ blood vessel development	4.59E−10	1.36E−06
GO:0048514 ~ blood vessel morphogenesis	5.91E−10	1.76E−06
GO:0001525 ~ angiogenesis	6.13E−10	1.82E−06
GO:0009611 ~ response to wounding	4.55E−09	1.35E−05
GO:0007155 ~ cell adhesion	9.76E−09	2.90E−05
GO:0022610 ~ biological adhesion	9.76E−09	2.90E−05
GO:0007242 ~ intracellular signaling cascade	6.29E−07	0.001869
GO:0019932 ~ second-messenger-mediated signaling	1.40E−06	0.004152
GO:0010033 ~ response to organic substance	1.41E−06	0.004182
GO:0055066 ~ di-, tri-valent inorganic cation homeostasis	1.13E−05	0.033116

As shown in Additional file 1: Figure S8, key up-regulated receptors including CXCR4, TEK, FGFR1, and KDR are essential to angiogenesis and other regeneration-associated signaling pathways. In the GO category *response to wounding* (Additional file 1: Figure S9), many genes that were up-regulated, such as NOTCH1, NOTCH3, TIMP3, VWF, ADAM15, GAS6, IGFBP1, and TM4SF4, also belong to the GO categories *wound healing* and *tissue regeneration*. Cell adhesion-related GO categories were found to be significantly up-regulated in B4 relative to PreG (Table 5; Additional file 1: Figure S10).

### Sub-population B5: Forty GO/KEGG categories found to differ from PreG

Sub-population B5 was found to be the most distinctive fraction relative to PreG at the gene expression level. Forty GO/KEGG categories were significantly different from PreG (Table 6). Up-regulated categories include *cell adhesion*, *angiogenesis* (Additional file 1: Figure S11), *branching morphogenesis of a tube* (Additional file 1: Figure S12), *cell morphogenesis, regulation of epithelial cell proliferation* (Additional file 1: Figure S13), *blood vessel development, blood vessel morphogenesis, vasculature development, cellular component morphogenesis, cell morphogenesis, cell morphogenesis involved in differentiation, tube morphogenesis, cell projection organization and morphogenesis, cell part morphogenesis, morphogenesis of a branching structure, developmental maturation* and *response to nutrient*. Most of these significantly up-regulated GO/KEGG categories are clearly involved in catalyzing reparative, restorative, or regenerative outcomes.

**Table 6:** GO categories and KEGG pathways that were significantly up-regulated in B5 by comparing to PreG

Category	Term	*P* value	Bonferroni adjusted P
GOTERM_BP_FAT	GO:0022610 ~ biological adhesion	1.36E−14	4.62E−11
	GO:0007155 ~ cell adhesion	1.36E−14	4.62E−11
	GO:0035295 ~ tube development	7.52E−10	2.57E−06
	GO:0001525 ~ angiogenesis	2.88E−09	9.84E−06
	GO:0001568 ~ blood vessel development	9.91E−09	3.38E−05
	GO:0048754 ~ branching morphogenesis of a tube	1.46E−08	5.00E−05
	GO:0048514 ~ blood vessel morphogenesis	1.57E−08	5.37E−05
	GO:0001944 ~ vasculature development	2.29E−08	7.82E−05
	GO:0032989 ~ cellular component morphogenesis	3.12E−08	1.06E−04
	GO:0000902 ~ cell morphogenesis	6.13E−08	2.09E−04
	GO:0000904 ~ cell morphogenesis involved in differentiation	1.24E−07	4.24E−04
	GO:0035239 ~ tube morphogenesis	1.31E−07	4.46E−04
	GO:0030030 ~ cell projection organization	1.49E−07	5.07E−04
	GO:0048858 ~ cell projection morphogenesis	2.42E−07	8.25E−04
	GO:0016337 ~ cell–cell adhesion	2.79E−07	9.52E−04
	GO:0048812 ~ neuron projection morphogenesis	5.46E−07	0.001862
	GO:0032990 ~ cell part morphogenesis	6.75E−07	0.0023
	GO:0048666 ~ neuron development	7.48E−07	0.002549
	GO:0033273 ~ response to vitamin	7.72E−07	0.002632
	GO:0001763 ~ morphogenesis of a branching structure	1.06E−06	0.003597
	GO:0021700 ~ developmental maturation	1.20E−06	0.004074
	GO:0031175 ~ neuron projection development	2.56E−06	0.008704
	GO:0050678 ~ regulation of epithelial cell proliferation	6.75E−06	0.022778
	GO:0007584 ~ response to nutrient	1.49E−05	0.049576
KEGG	rno04514:Cell adhesion molecules (CAMs)	5.89E−05	0.009201

The canonical WNT/β-catenin signaling pathway is significantly down-regulated relative to PreG (Table 7; Additional file 1: Figure S14). As observed with B4, the key pro-angiogenic receptors CXCR4, TEK, and KDR are prominently up-regulated in B5. In addition, the pro-angiogenic ligands PGF, ANGPT2, ANGPT4, VEGFA, and PDGFA are significantly up-regulated in B5 relative to PreG. Activation of the regulation of GO category *epithelial cell proliferation* through the TGF-β1 signaling pathway is consistent with a potential role for SRC in the promotion of host tubular epithelial cell proliferation (see Additional file 1: Figure S13).

**Table 7:** GO categories and KEGG pathways that were significantly down-regulated in B5 by comparing to PreG

Category	Term	Raw-*P*	Bonferroni adjusted-*P*
GOTERM_BP_FAT	GO:0044265 ~ cellular macromolecule catabolic process	5.44E−08	1.79E−04
	GO:0007242 ~ intracellular signaling cascade	2.07E−07	6.83E−04
	GO:0051603 ~ proteolysis involved in cellular protein catabolic process	5.90E−07	0.001943
	GO:0044257 ~ cellular protein catabolic process	7.90E−07	0.002602
	GO:0043632 ~ modification-dependent macromolecule catabolic process	1.48E−06	0.004857
	GO:0019941 ~ modification-dependent protein catabolic process	1.48E−06	0.004857
	GO:0015031 ~ protein transport	1.99E−06	0.006541
	GO:0030163 ~ protein catabolic process	2.23E−06	0.007313
	GO:0045184 ~ establishment of protein localization	2.78E−06	0.009125
	GO:0009057 ~ macromolecule catabolic process	3.47E−06	0.011365
KEGG	rno04120:Ubiquitin-mediated proteolysis	1.17E−07	2.02E−05
	rno04310:Wnt signaling pathway	8.10E−06	0.001392
	rno05200:Pathways in cancer	8.19E−06	0.001408
	rno05222:Small cell lung cancer	2.02E−05	0.003468
	rno04530:Tight junction	2.11E−04	0.035682

## Conclusions

1. *SRC component characterization* Transcriptomic profiling of B1–B5 confirms functional analysis of distinctive reparative, restorative, or regenerative outcomes associated with cell transplantation of each of B1–B5 in rodent models of chronic kidney disease. Iodixanol gradient density centrifugation of PreG renal cells generates 5 unique subpopulations with distinctive transcriptional signatures.
2. *Sub-fraction B1* GO/KEGG categories associated with cell cycle and transcriptional control were down-regulated relative to PreG. GO/KEGG categories associated with inflammation were up-regulated relative to PreG
3. *Sub-fraction B2* Most similar to PreG. Only GO/KEGG categories associated with ECM–receptor interaction are significantly down-regulated relative to PreG.
4. *Sub-fraction B3* GO/KEGG categories associated with immune response were significantly up-regulated relative to PreG. GO/KEGG categories associated with regulation of actin polymerization were significantly down-regulated relative to PreG
5. *Sub-fraction B4* GO/KEGG categories associated with repair, restoration, or regeneration and cell adhesion were significantly up-regulated relative to PreG.
6. *Sub-population B5* The most distinctive sub-population relative to PreG. Forty GO/KEGG categories found to be significantly different from PreG including multiple categories unambiguously associated with repair, restoration, or regeneration.

## Discussion

SRC are manufactured through iodixanol gradient centrifugation of a PreG population principally composed of renal tubular epithelial cells. The standard for SRC separation and reconstitution is entirely biophysical, based on the identification, isolation, and recombination of bands of defined density following gradient centrifugation. No attempt is made to achieve a predefined cellular composition expressing certain cell surface or other markers. In this, SRC are distinct from other cell-based therapeutics currently under development. As SRC is an autologous product, each patient biopsy results in an unique product, also composed principally of tubular epithelial cells. Although manufacturing conditions including media composition may well bias for or against certain cell sub-populations, no attempt is made here to control for selection bias secondary to media formulation. Following gradient centrifugation, B1 is selectively eliminated and B2–B5 collapsed together to create the final product.

Transcriptomic profiling is consistent with SRC representing a pro-regenerative cell population capable of catalyzing functional rescue of aspects of the disease condition in rodent models of chronic kidney disease. Specifically, the transcriptomic analysis of SRC component cell populations presented in this manuscript suggests that B1 subpopulations present significantly less proliferative and regenerative potential, demonstrated by the down-regulation of genetic networks associated with cell cycle and transcriptional control. These data are consistent with the observed absence of functional impact by B1 cells on renal pathophysiology in rodent models of CKD [6, 7, 10]. The transcriptomic profile of B2 was found to be similar to PreG at the pathway level. No GO/KEGG categories were identified as significantly up-regulated. Only one pathway, ECM–receptor interaction (rno04512), was found to be significantly down-regulated. It is well known that excessive deposition of extracellular matrix (ECM) components causes tissue fibrosis [18–20]. Renal fibrosis is the hallmark of disease progression from chronic kidney disease (CKD) to end-stage kidney disease. The lower expression level of ECM proteins in B2 may contribute to the lower level of fibrosis in B2 treated CKD rats, which may in turn explain the therapeutic benefits previously observed in B2 when compared to PreG [6, 7, 10].

In contrast, RHAMM expression was specifically up-regulated in B2/PreG. Expression of the hyaluronic acid (HA) receptor CD168/RHAMM has been directly linked to regenerative bioactivity in a number of in vitro and in vivo model systems. In addition, RHAMM has been shown to promote angiogenesis and cell motility; the increased expression level of RHAMM could be beneficial for tissue regeneration [21–26]. For B3, KEGG and GO analysis found a significant increase in the humoral immune response. It is well established that multiple components of both cellular and humoral immune systems contribute to reparative, restorative, or regenerative outcomes in multiple organs and tissues, including limbs, skeletal muscle, heart, and nervous system [27].

Consistent with its established therapeutic bioactivity in vivo, multiple regeneration-related GO categories, including vasculature development, blood vessel development, blood vessel morphogenesis, angiogenesis, and response to wounding, were found to be significantly up-regulated in B4 over PreG. Angiogenesis (Additional file 1: Figure S8) is central to regeneration, in the kidney, heart, and multiple other organs and tissues [28–31]. Sub-population B5 was found to be the most distinctive fraction relative to PreG at the gene expression level. Up-regulated categories include cell adhesion, angiogenesis, branching morphogenesis of a tube, cell morphogenesis, regulation of epithelial cell proliferation, blood vessel development, blood vessel morphogenesis, vasculature development, cellular component morphogenesis, cell morphogenesis, cell morphogenesis involved in differentiation, tube morphogenesis, cell projection organization and morphogenesis, cell part morphogenesis, morphogenesis of a branching structure, developmental maturation, and response to nutrients, etc. Most of these significantly up-regulated GO/KEGG categories are clearly involved in catalyzing regenerative outcomes.

SRC manufacture applies a 24-h period of exposure to hypoxia (2% oxygen) to the expanded cell population prior to gradient centrifugation. Hypoxic preconditioning has been used to promote the pro-regenerative characteristics of mesenchymal stem cells in preclinical rodent models of renal disease [32]. Consistent with the results presented in this manuscript, genome-wide transcriptional profiling of the effect of hypoxic preconditioning on human mesenchymal stem cells identified altered expression of genes involved in inflammation, immune function, cell survival, migration and proliferation as well as vasculogenesis and angiogenesis [33]. More broadly, genome-wide transcriptional profiling has been leveraged as a tool to monitor product quality and consistency during the manufacture of cell-based therapeutics including mesenchymal stem cells, induced pluripotent stem cells, macrophages, and cardiac progenitors [34–38]. A similar approach may be applied during SRC manufacture, with the expression of key regenerative gene networks serving as metrics of product character and potency [39, 40].

Taken together, these data suggest that SRC catalyze regenerative outcomes in the kidney in part by activating multiple transcriptomic networks associated with the promotion of regeneration while simultaneously suppressing alternate transcriptional pathways that may promote the continued development of renal disease and pathophysiology, see Additional file 1: Figure S17.

### Future perspectives

SRC is currently being directly evaluated in Phase II clinical trials in patients with type 2 diabetes and chronic kidney disease (www.prokidney.com) This trial is built on the success of a Phase I clinical trial of 7 type II diabetic patients with CKD3/4. SRC formulated and delivered in a hydrogel carrier (“REACT”) was shown to be safe and well-tolerated. As SRC transitions through the clinical translation pathway, a clear understanding of how observed clinical outcomes are mediated through cell bioactivity is essential. The current manuscript establishes a framework for a putative mechanism of action for SRC in rodent preclinical models. In future studies, these results will be extended and further developed using human SRC (Basu et al., manuscript in preparation). As described in [39, 40], mechanistic pathways identified through transcriptomic and other related methodologies may be leveraged for the development and application of specific potency assays for quantitation of SRC regenerative bioactivity.

## Supplementary Information

**Additional file 1.** **Figures S1–S16:** Interactomic analysis of transcripts in B1–B5 up- or down-regulated relative to PreG. **Figure S17:** Summary figure of transcriptomic signatures associated with each of bands B1–B5. SRC are assembled by reconstitution of B2–B5 as shown. 

## Data Availability

Please contact corresponding author at joydeep.basu@prokidney.com.

## Abbreviations

SRC: Selected renal cells
MOA: Mechanism of action
CKD: Chronic kidney disease
PreG: Pregradient
ECM: Extra-cellular matrix
EMT: Epithelial–mesenchymal transition
5/6N_*x*_: 5/6 Nephrectomy
ESRD: End-stage renal disease
ZSF1: *Zucker* Diabetic nephropathy
GO: Gene ontogeny
KEGG: Kyoto encyclopedia of genes and genomes
MSC: Mesenchymal stem cell
HA: Hyaluronic acid
