# Supplementary material for: Identification of functional pathways for regenerative bioactivity of selected renal cells
Source: Stem Cell Res Ther. 2022 Feb 17;13:72. doi: 10.1186/s13287-022-02713-6 (PMC8851708; doi:10.1186/s13287-022-02713-6)
Supplement: Supplementary file 1 — Additional file 1. Figures S1–S16: Interactomic analysis of transcripts in B1–B5 up- or down-regulated relative to PreG. Figure S17: Summary figure of transcriptomic signatures associated with each of bands B1–B5. SRC are assembled by reconstitution of B2–B5 as shown. [file 13287_2022_2713_MOESM1_ESM.pdf]

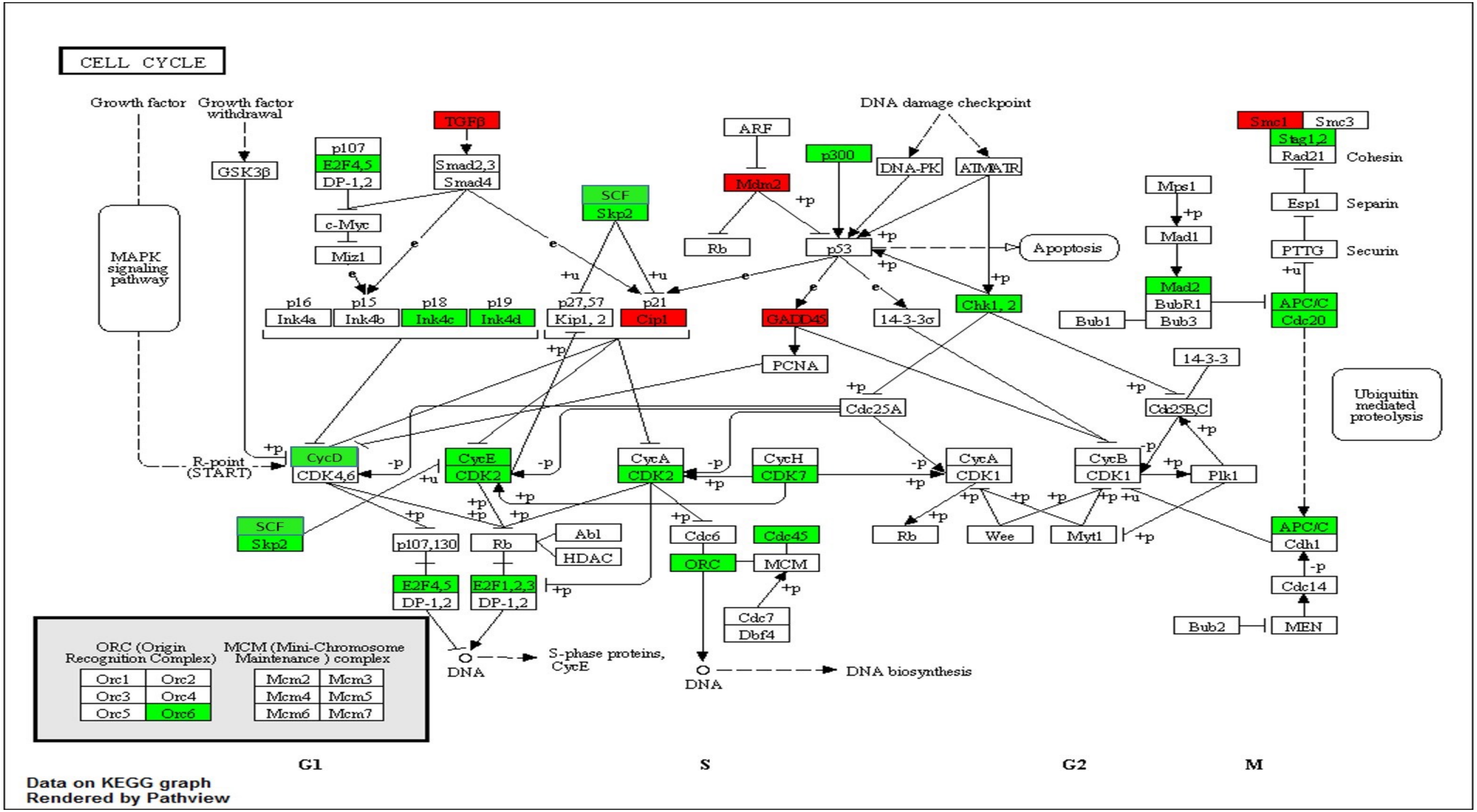

Figure 1: Cell cycle was significantly down-regulated (P = 0.042) in B1 by comparing to PreG. Genes that were up (red) or down (green) regulated in B1 were determined by paired t-test between B1 and PreG at P<0.05.

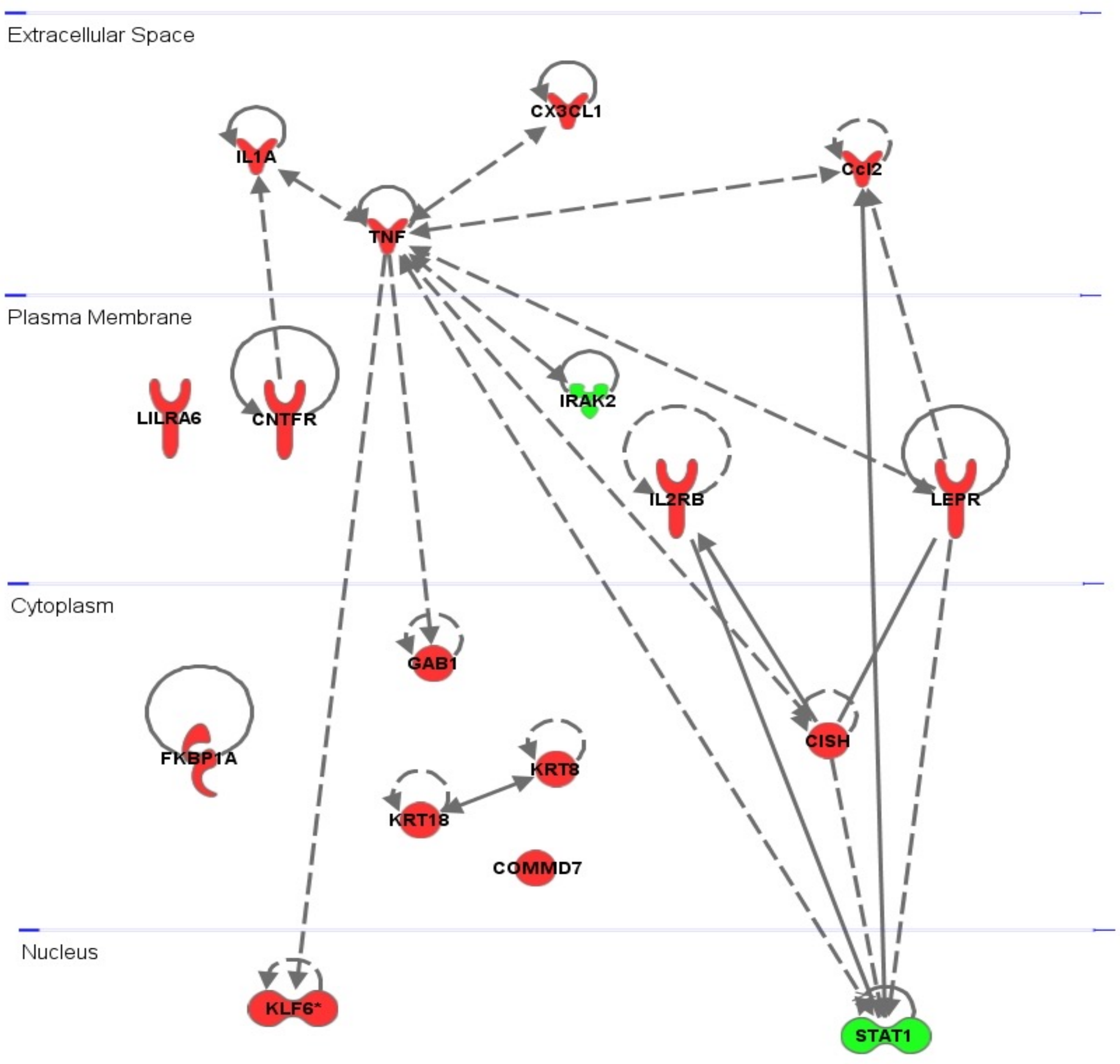

© 2000-2014 QIAGEN. All rights reserved.

Figure 2: Cytokine-mediated signaling pathway (GO:0019221) was significantly up-regulated (P = 0.023) in B1 by comparing to PreG. Genes that belong to this GO category and were differentially expressed between B1 and PreG are included in this figure. Differentially expressed genes were determined by paired t-test at P<0.05. Up-regulated genes are in red. Down-regulated genes are in green. A figure legend about molecule shapes and their connections is at http://ingenuity.force.com/ipa/articles/Feature_Description/Legend.

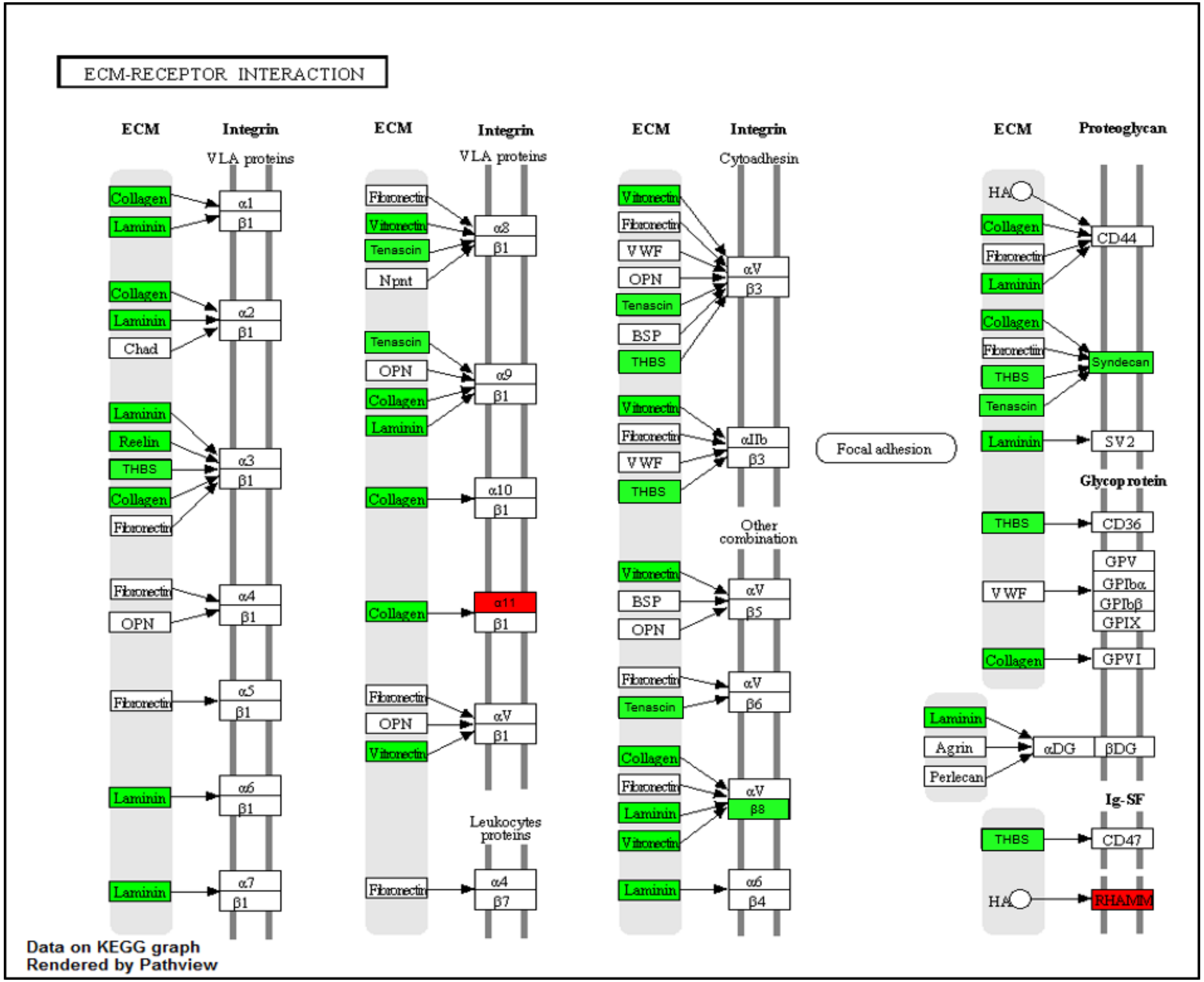

Figure 3: ECM-receptor interaction pathway was significantly down-regulated (P = 0.039) in B2 by comparing to PreG. Genes that were up (red) or down (green) regulated in B2 were determined by paired t-test between B2 and PreG at P<0.05.

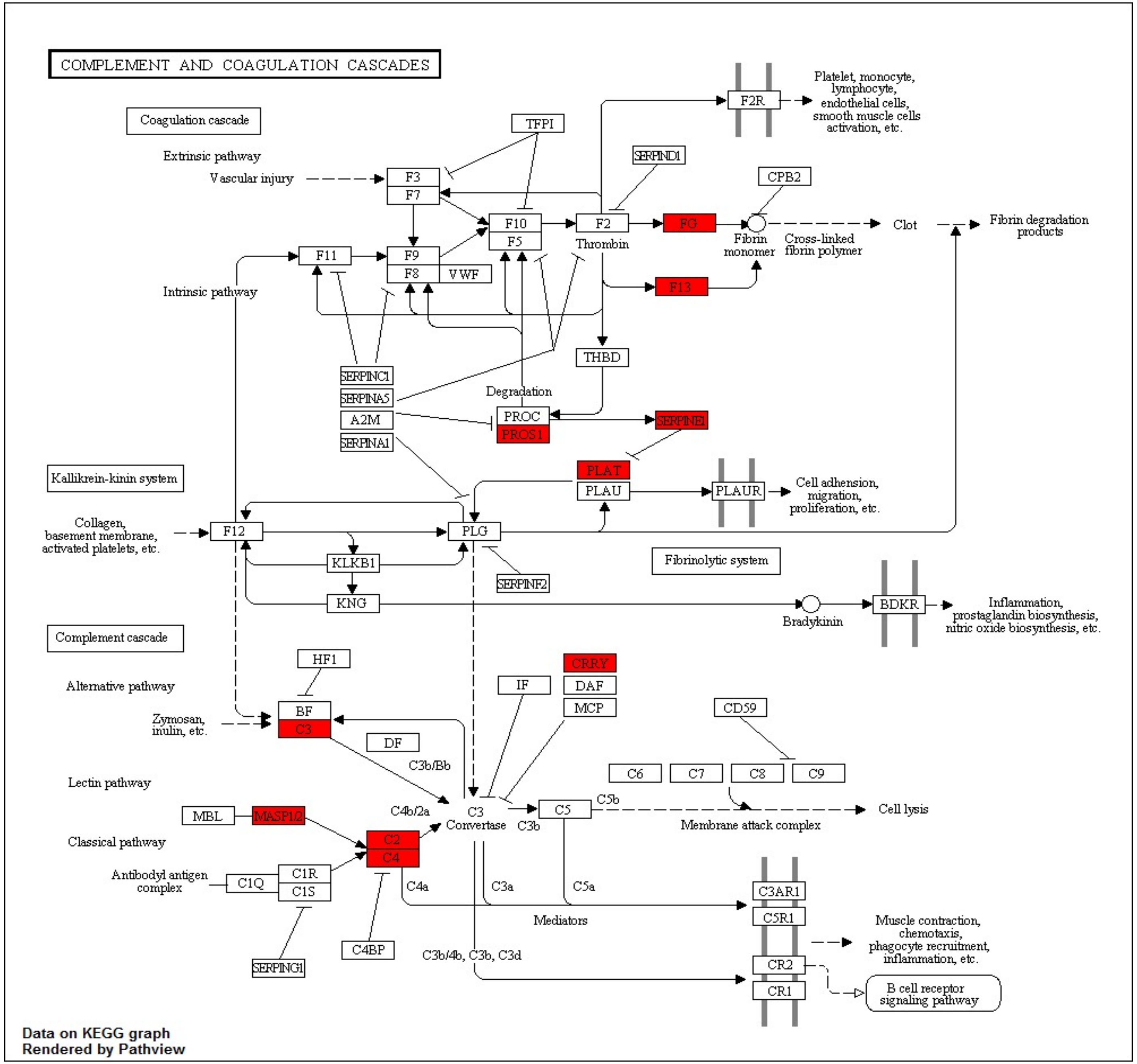

Figure 4 : Differentially expressed genes (P<0.05) in complement and coagulation cascades in B3 by comparing to PreG. Differentially expressed genes were determined by paired t-test between B3 and PreG at P<0.05. All of the differentially expressed genes in this pathway were found to be up-regulated (red) in B3.

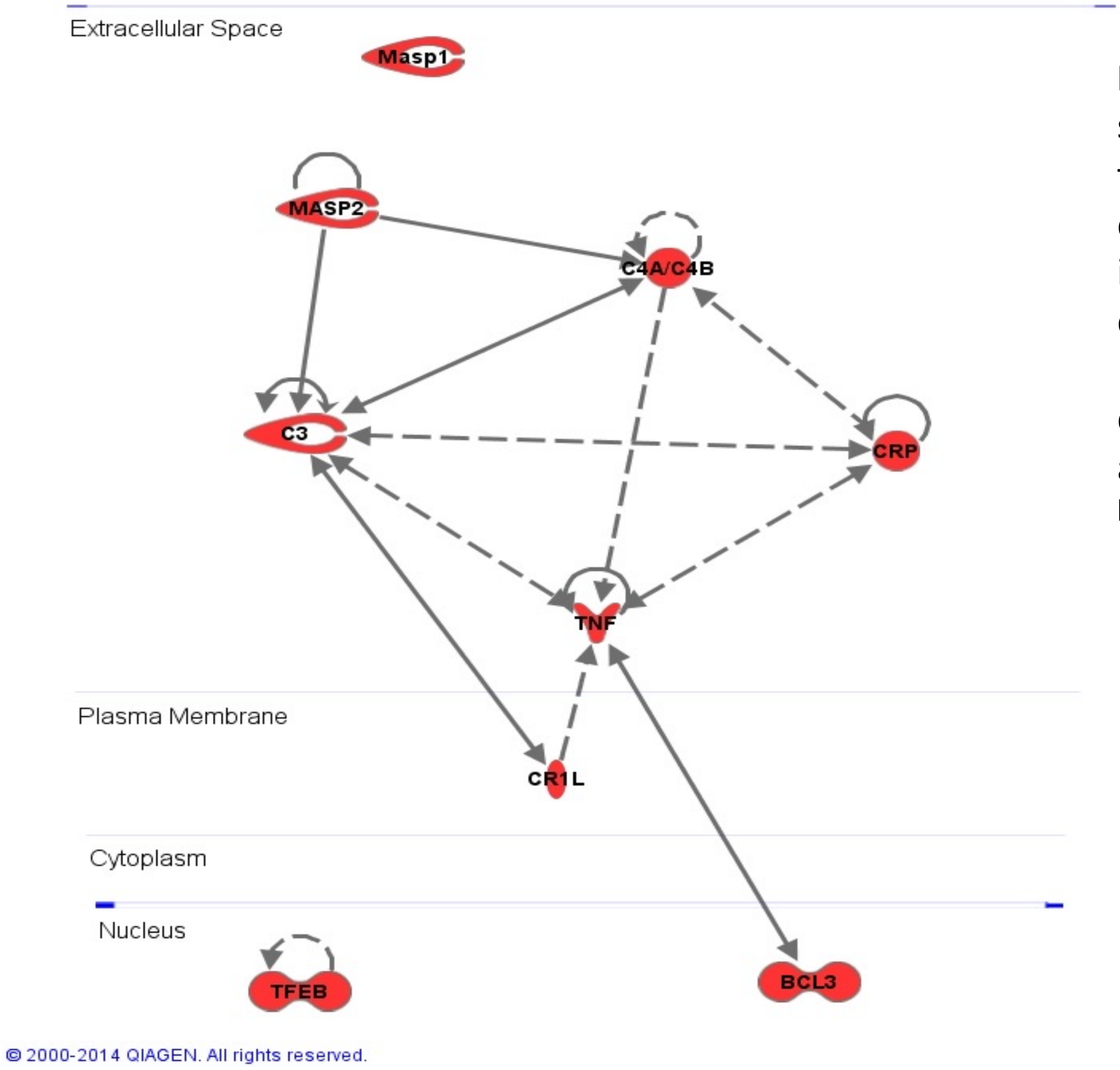

Figure 5: Humoral immune response (GO: 0006959) was significantly up-regulated (P = 0.025) in B3 by comparing to PreG. Genes that belong to this GO category and were differentially expressed between B3 and PreG are included in this figure. Differentially expressed genes were determined by paired t-test at P<0.05. Up-regulated genes are in red. None of the genes in this GO category were down-regulated. A figure legend about molecule shapes and their connections is at http://ingenuity.force.com/ipa/articles/Feature_Description/Legend.

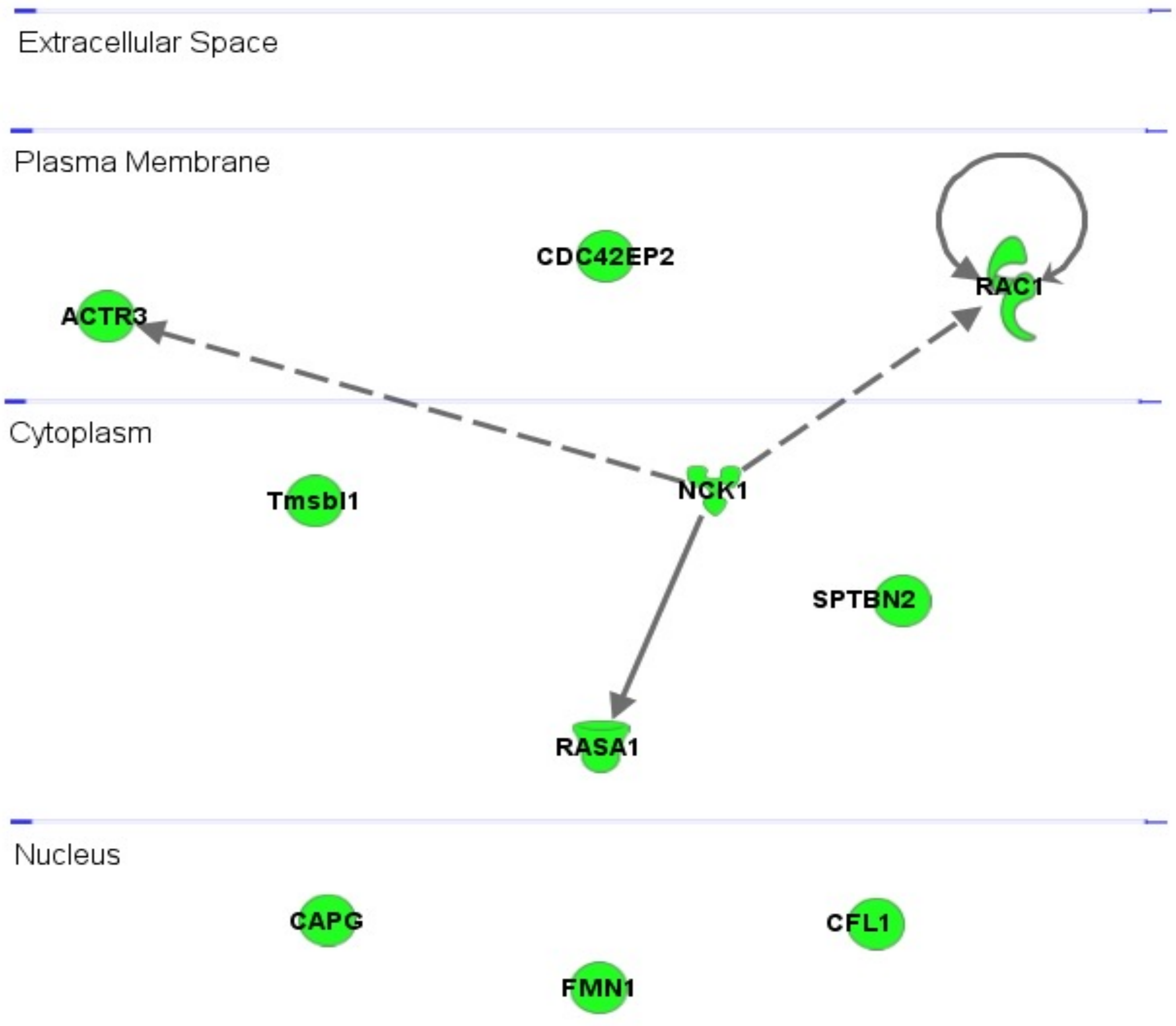

© 2000-2014 QIAGEN. All rights reserved.

Figure 6: Regulation of actin filament length (GO:0030832) was significantly down-regulated ($P = 0.00522$) in B3 by comparing to PreG. Genes that belong to this GO category and were differentially expressed between B3 and PreG are included in this figure. Differentially expressed genes were determined by paired t-test at $P<0.05$. Down-regulated genes are in green. None of the genes in this GO category were up-regulated. A figure legend about molecule shapes and their connections is at http://ingenuity.force.com/ipa/articles/Feature_Description/Legend.

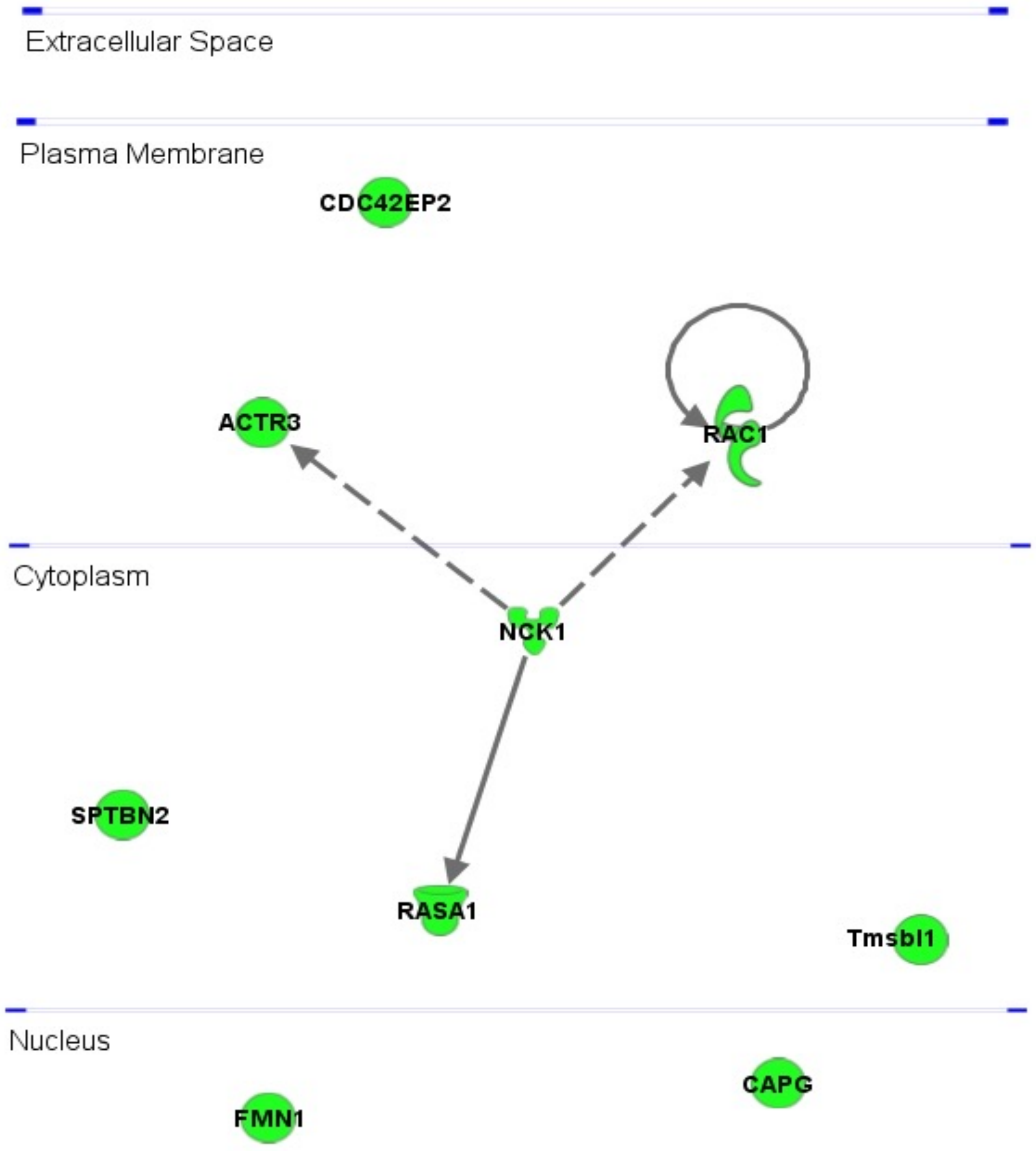

© 2000-2014 QIAGEN. All rights reserved.

Figure 7: Regulation of actin filament polymerization (GO: 0030833) was significantly down-regulated (P = 0.018633) in B3 by comparing to PreG. Genes that belong to this GO category and were differentially expressed between B3 and PreG are included in this figure. Differentially expressed genes were determined by paired t-test at P<0.05. Down-regulated genes are in green. None of the genes in this GO category were up-regulated. A figure legend about molecule shapes and their connections is at http://ingenuity.force.com/ipa/articles/Feature_Description/Legend.

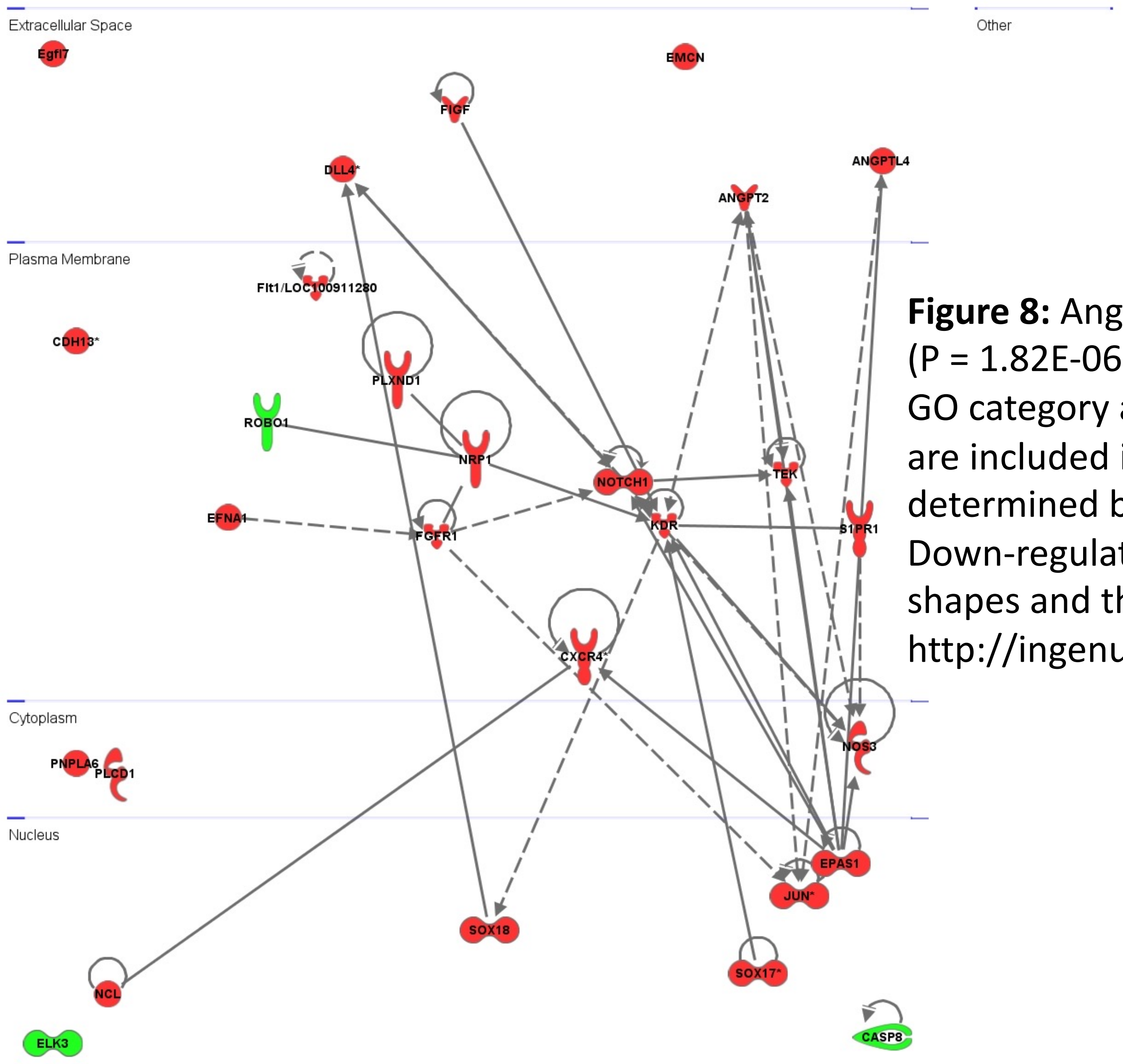

© 2000-2014 QIAGEN. All rights reserved.

Figure 8: Angiogenesis (GO: 0001525) was significantly up-regulated (P = 1.82E-06) in B4 by comparing to PreG. Genes that belong to this GO category and were differentially expressed between B4 and PreG are included in this figure. Differentially expressed genes were determined by paired t-test at $P<0.05$. Up-regulated genes are in red. Down-regulated genes are in green. A figure legend about molecule shapes and their connections is at http://ingenuity.force.com/ipa/articles/Feature_Description/Legend.

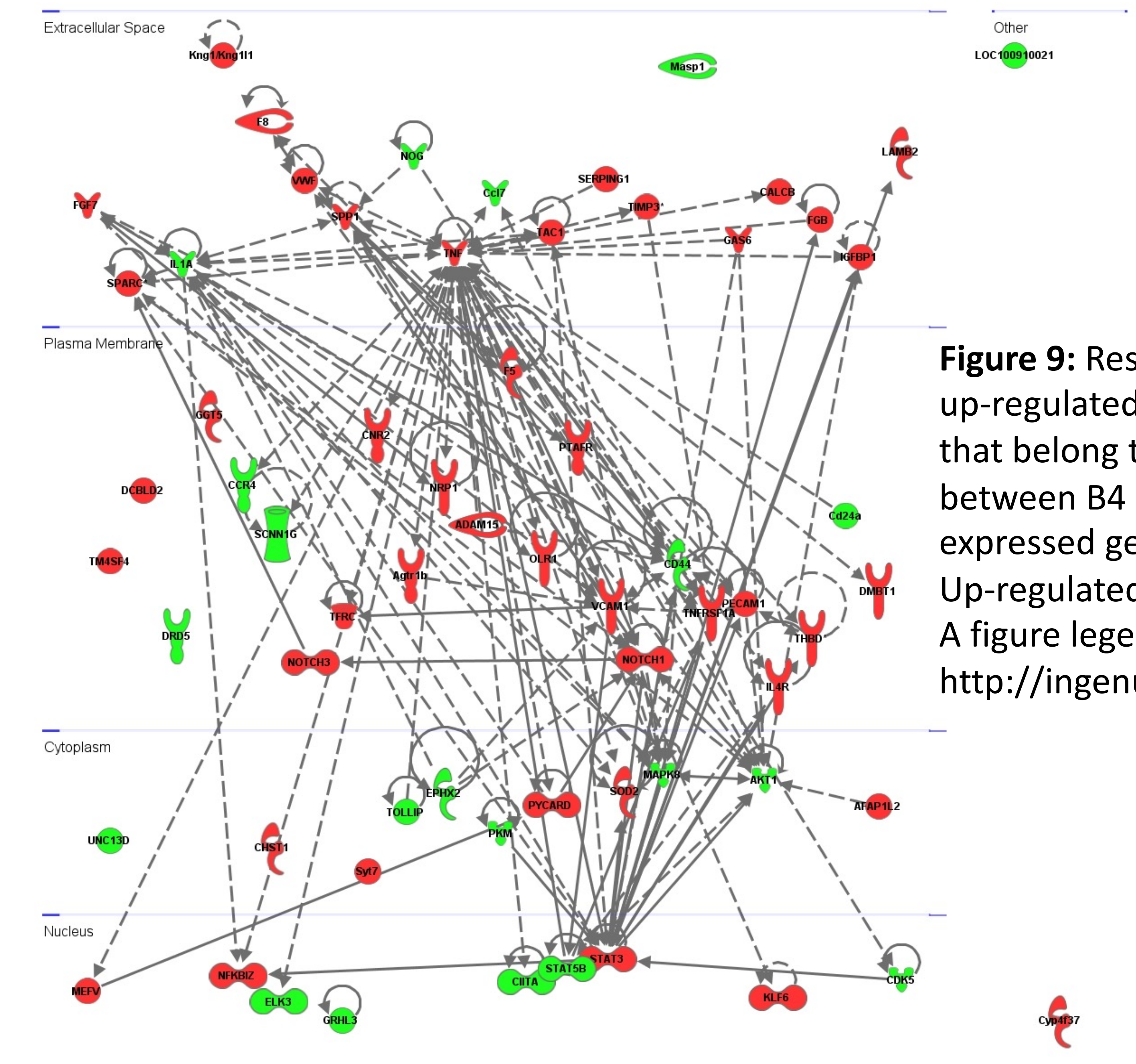

Figure 9: Response to wounding (GO:0009611) was significantly up-regulated (P = 1.35E-05) in B4 by comparing to PreG. Genes that belong to this GO category and were differentially expressed between B4 and PreG are included in this figure. Differentially expressed genes were determined by paired t-test at P<0.05. Up-regulated genes are in red. Down-regulated genes are in green. A figure legend about molecule shapes and their connections is at http://ingenuity.force.com/ipa/articles/Feature_Description/Legend.

© 2000-2014 QIAGEN. All rights reserved.

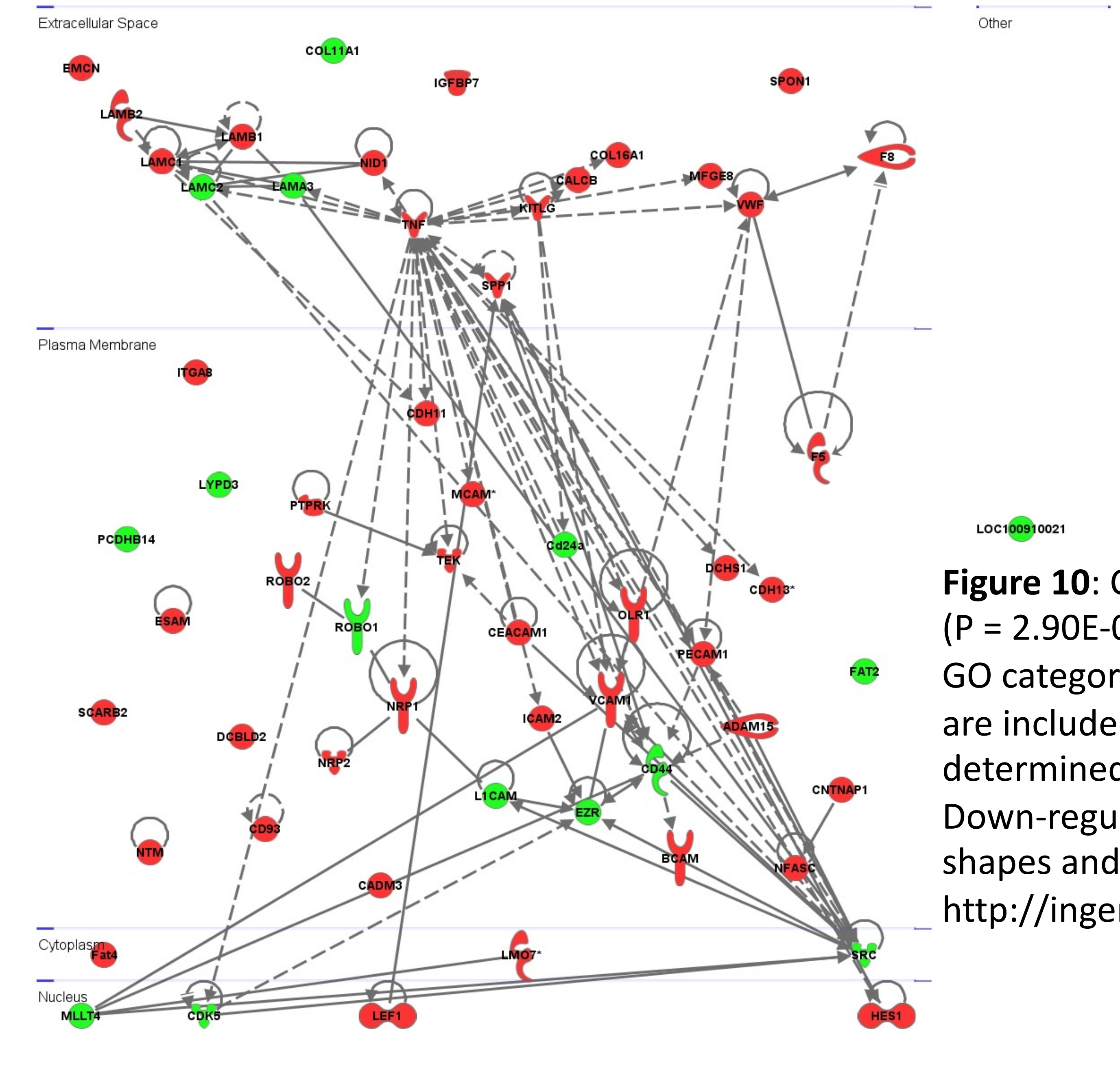

© 2000-2014 QIAGEN. All rights reserved.

Figure 10: Cell adhesion (GO: 0007155) was significantly up-regulated (P = 2.90E-05) in B4 by comparing to PreG. Genes that belong to this GO category and were differentially expressed between B4 and PreG are included in this figure. Differentially expressed genes were determined by paired t-test at P<0.05. Up-regulated genes are in red. Down-regulated genes are in green. A figure legend about molecule shapes and their connections is at http://ingenuity.force.com/ipa/articles/Feature_Description/Legend.

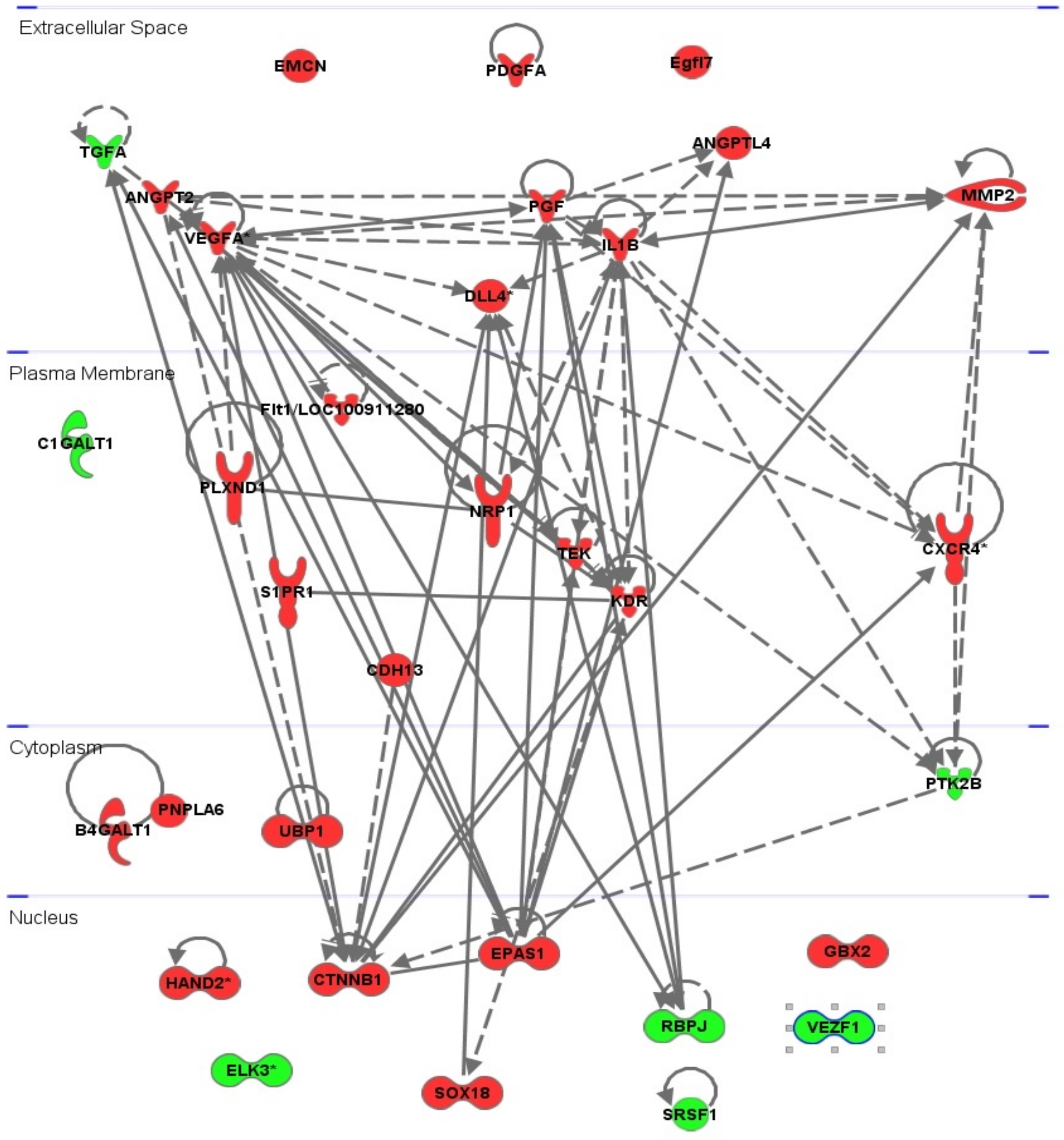

© 2000-2014 QIAGEN. All rights reserved.

Figure 11: Angiogenesis (GO: 0001525) was significantly up-regulated (P = 9.84E-06) in B5 when compared to PreG. Genes that belong to this GO category and were differentially expressed between B5 and PreG are included in this figure. Differentially expressed genes were determined by paired t-test at P<0.05. Up-regulated genes are in red. Down-regulated genes are in green. A figure legend about molecule shapes and their connections is at http://ingenuity.force.com/ipa/articles/Feature_Description/Legend.

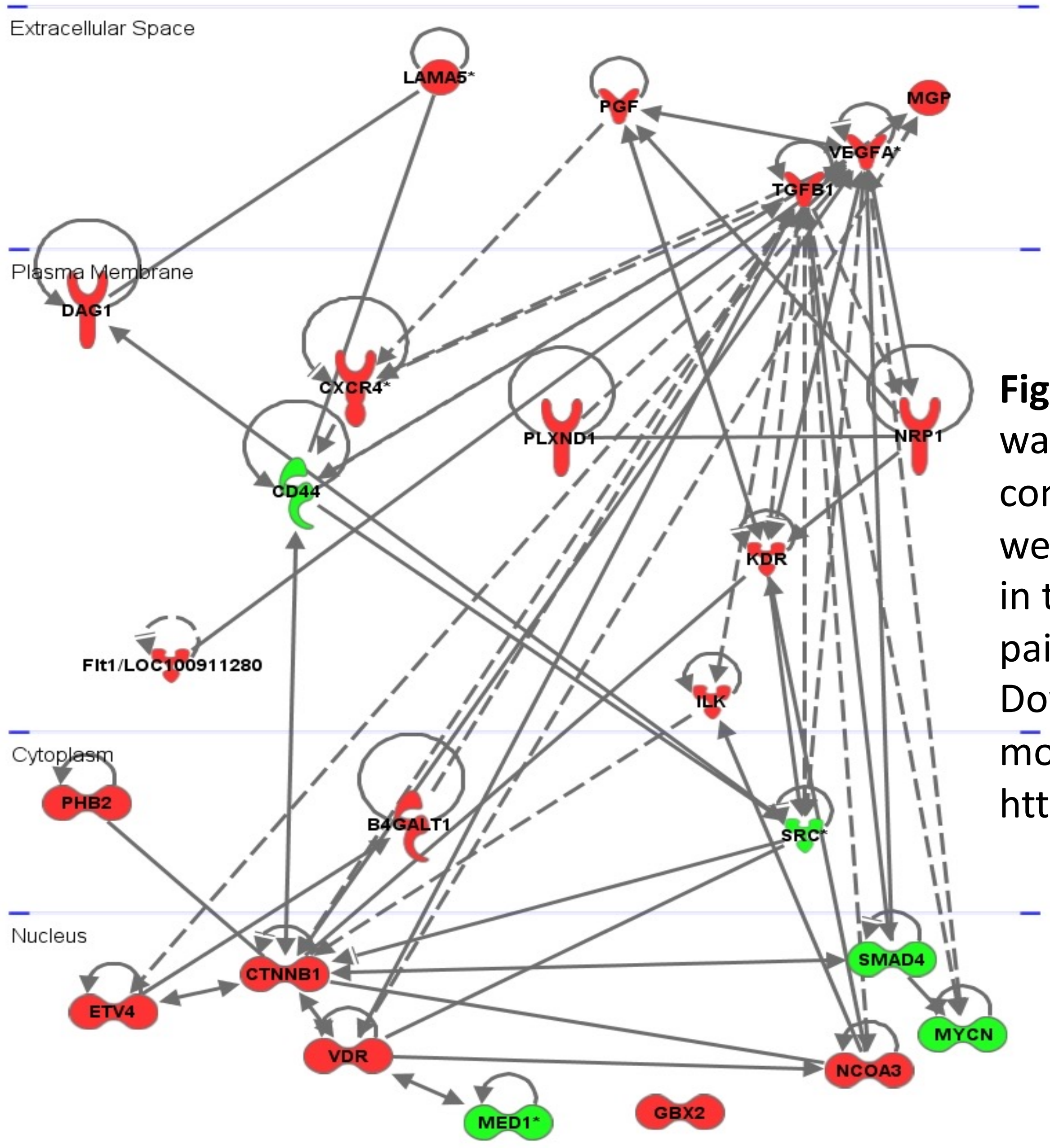

© 2000-2014 QIAGEN. All rights reserved.

Figure 12: Branching morphogenesis of a tube (GO: 0048754) was significantly up-regulated (P = 5.00E-05) in B5 when compared to PreG. Genes that belong to this GO category and were differentially expressed between B5 and PreG are included in this figure. Differentially expressed genes were determined by paired t-test at P<0.05. Up-regulated genes are in red. Down-regulated genes are in green. A figure legend about molecule shapes and their connections is at http://ingenuity.force.com/ipa/articles/Feature_Description/Legend.

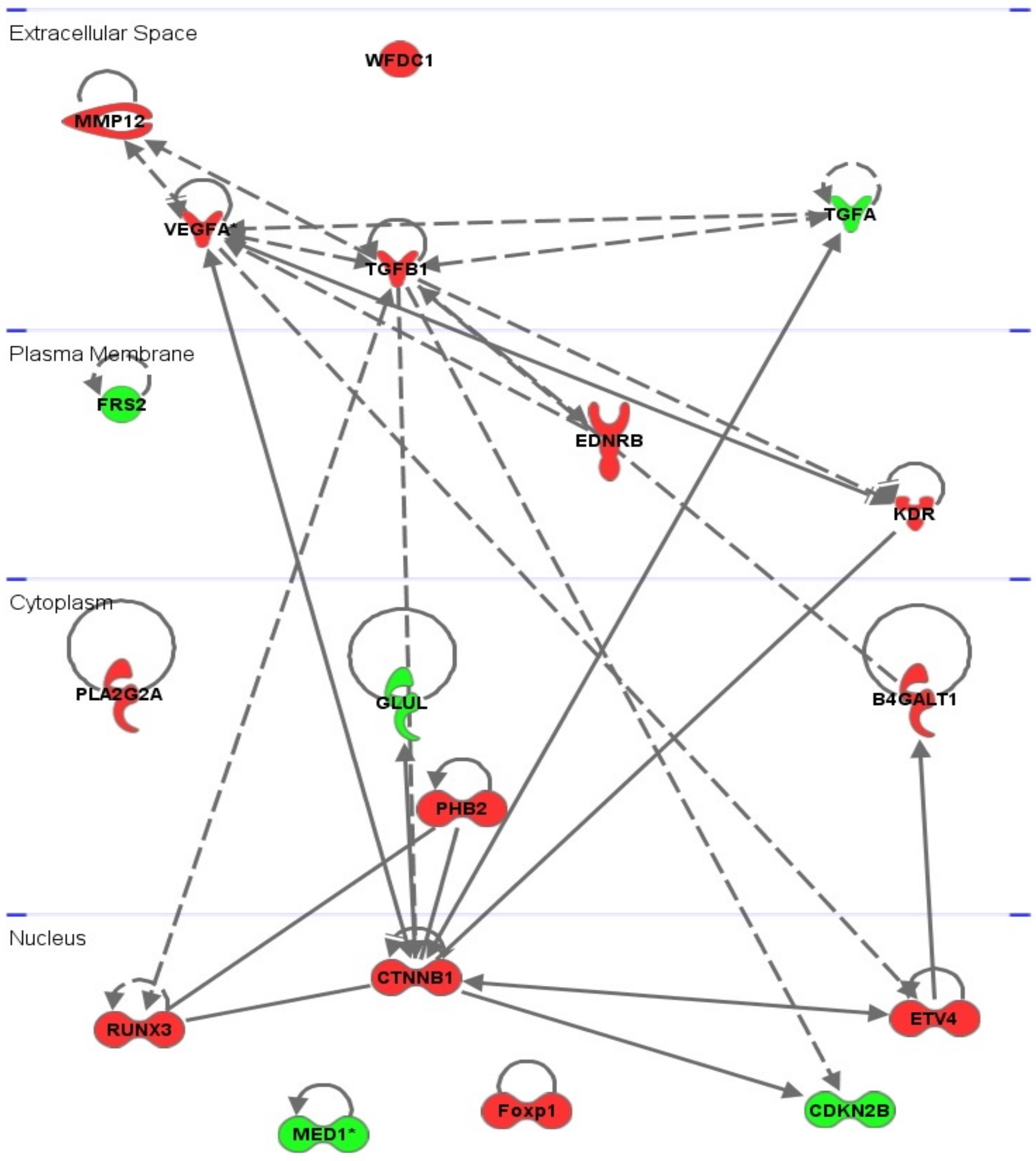

© 2000-2014 QIAGEN. All rights reserved.

Figure 13: Regulation of epithelial cell proliferation (GO:0050678) was significantly up-regulated (P = 0.022778) in B5 when compared to PreG. Genes that belong to this GO category and were differentially expressed between B5 and PreG are included in this figure. Differentially expressed genes were determined by paired t-test at P<0.05. Up-regulated genes are in red. Down-regulated genes are in green. A figure legend about molecule shapes and their connections is at http://ingenuity.force.com/ipa/articles/Feature_Description/Legend.

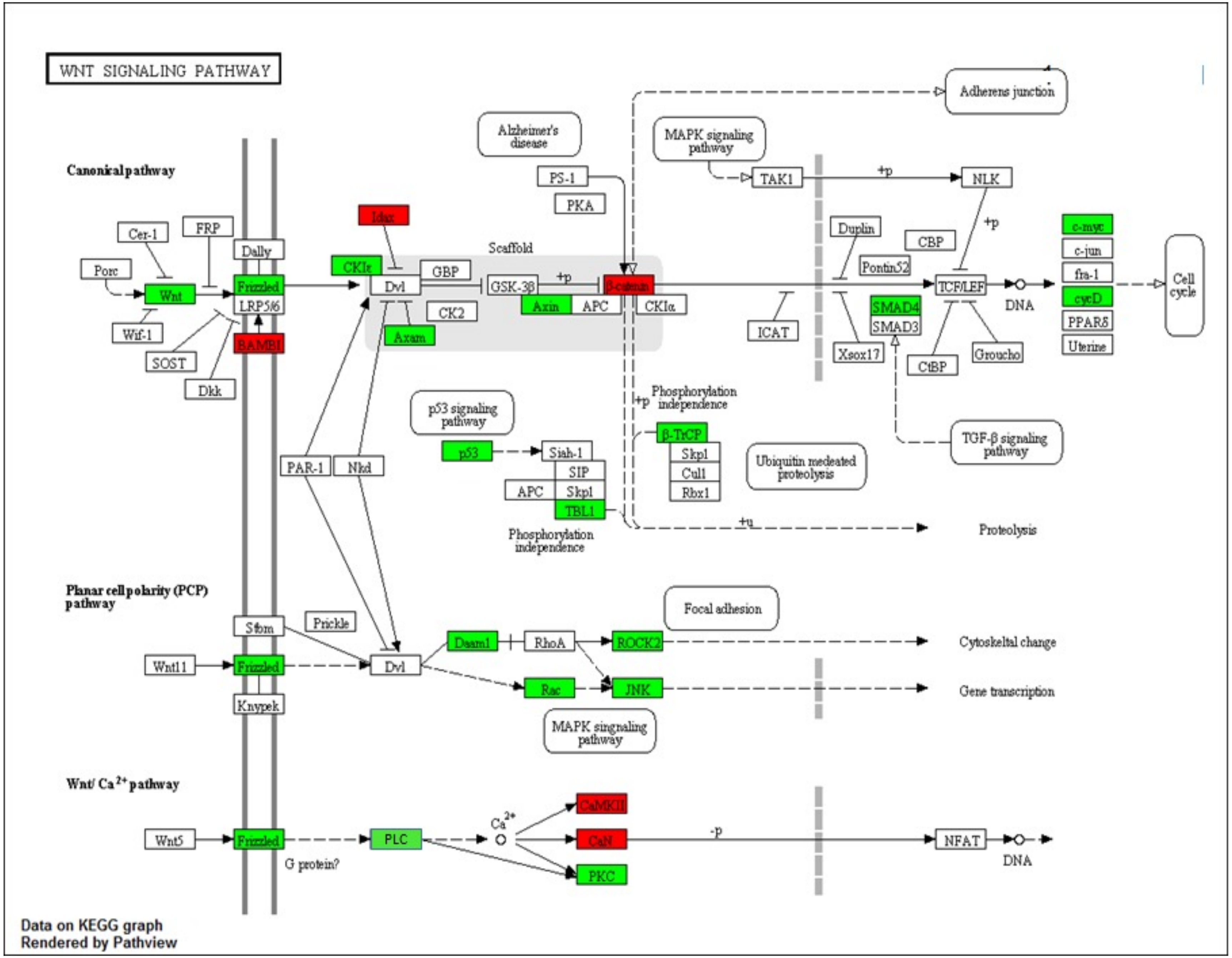

Figure 14: Wnt signaling pathway was significantly down-regulated ($P = 0.001392$) in B5 by comparing to PreG. Genes that were up (red) or down (green) regulated in B5 were determined by paired t-test between B5 and PreG at $P<0.05$.

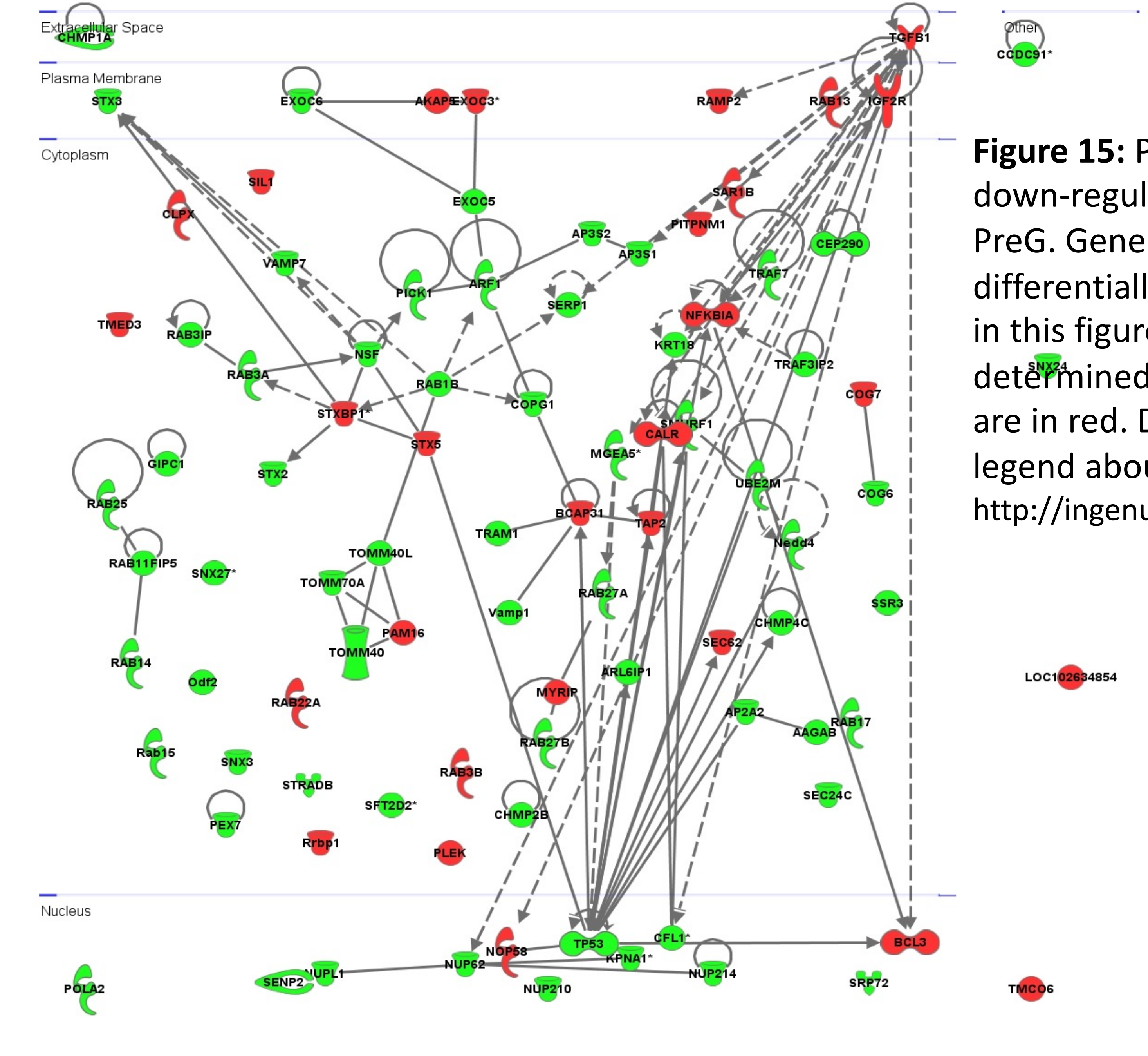

© 2000-2014 QIAGEN. All rights reserved.

Figure 15: Protein transport (GO: 0015031) was significantly down-regulated (P = 0.006541) in B5 when compared to PreG. Genes that belong to this GO category and were differentially expressed between B5 and PreG are included in this figure. Differentially expressed genes were determined by paired t-test at P<0.05. Up-regulated genes are in red. Down-regulated genes are in green. A figure legend about molecule shapes and their connections is at http://ingenuity.force.com/ipa/articles/Feature_Description/Legend.

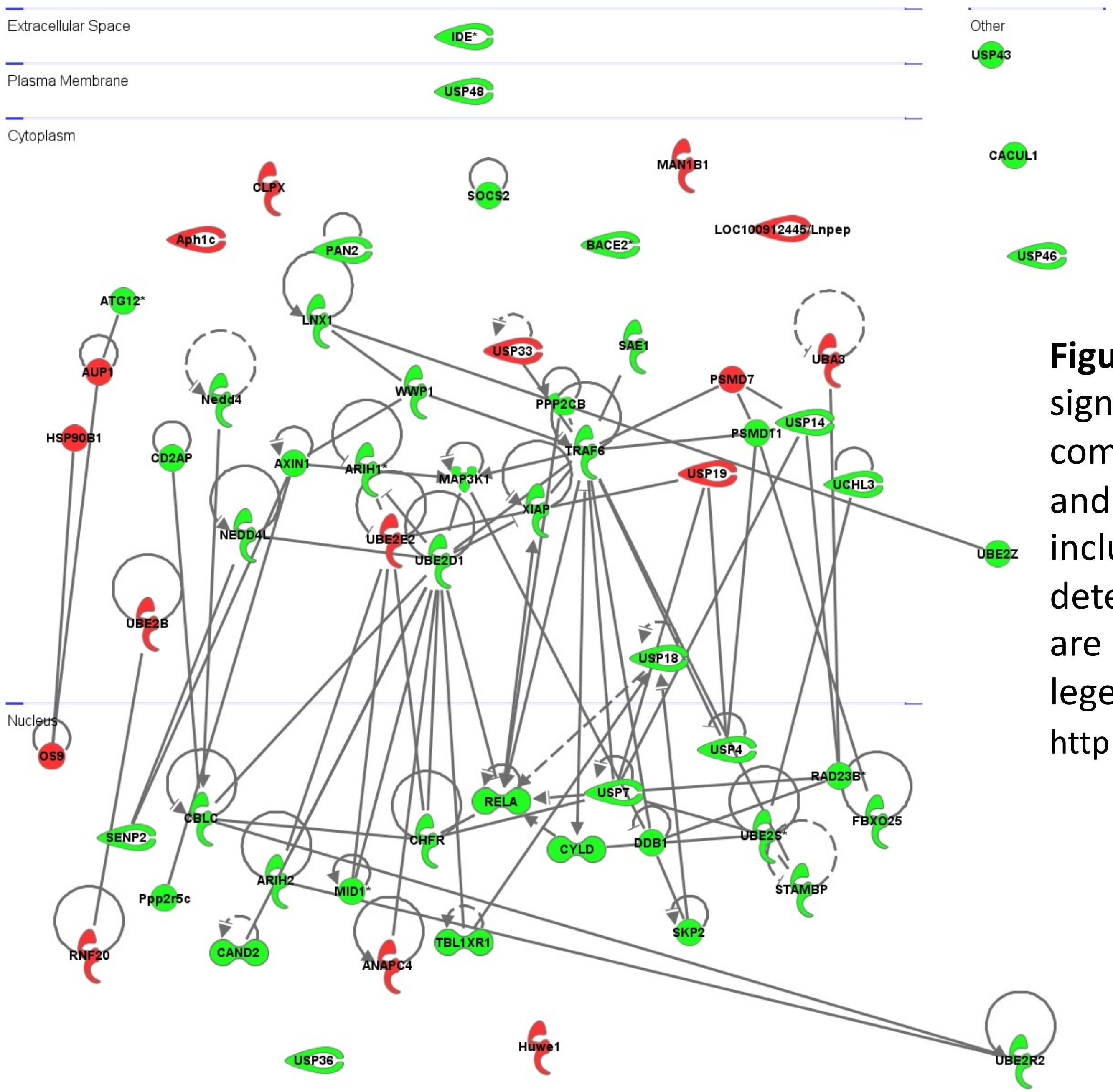

© 2000-2014 QIAGEN. All rights reserved.

Figure 16: Protein catabolic process (GO: 0030163) was significantly down-regulated (P = 0.007313) in B5 when compared to PreG. Genes that belong to this GO category and were differentially expressed between B5 and PreG are included in this figure. Differentially expressed genes were determined by paired t-test at P<0.05. Up-regulated genes are in red. Down-regulated genes are in green. A figure legend about molecule shapes and their connections is at http://ingenuity.force.com/ipa/articles/Feature_Description/Legend.

Figure 17

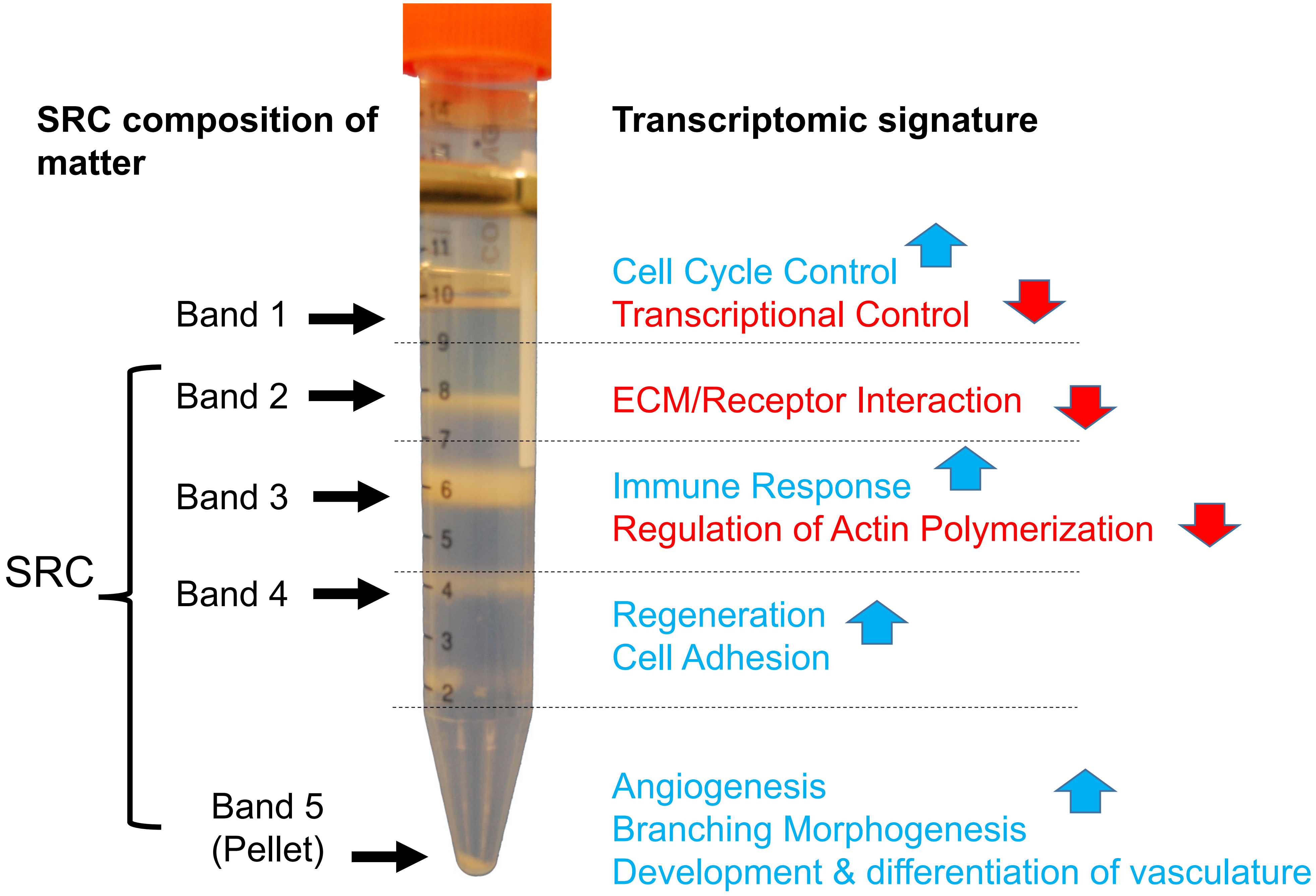
SRC composition of matter
Transcriptomic signature
Band 1
Band 2
Band 3
Band 4
Band 5
(Pellet)
SRC
Cell Cycle Control
Transcriptional Control
ECM/Receptor Interaction
Immune Response
Regulation of Actin Polymerization
Regeneration
Cell Adhesion
Angiogenesis
Branching Morphogenesis
Development & differentiation of vasculature
